# Awareness, willingness and barriers to HIV Self-testing (HIVST) among Men who Have Sex with Men (MSM) in Brazil, Mexico, and Peru: A web-based cross-sectional study

**DOI:** 10.1371/journal.pgph.0000678

**Published:** 2022-07-29

**Authors:** Oliver A. Elorreaga, Thiago S. Torres, E. Hamid Vega-Ramirez, Kelika A. Konda, Brenda Hoagland, Marcos Benedetti, Cristina Pimenta, Dulce Diaz-Sosa, Rebeca Robles-Garcia, Beatriz Grinsztejn, Carlos F. Caceres, Valdilea G. Veloso

**Affiliations:** 1 Centro de Investigación Interdisciplinaria en Sexualidad Sida y Sociedad, UPCH, Lima, Peru; 2 Instituto Nacional de Infectologia Evandro Chagas, Fundação Oswaldo Cruz (INI-Fiocruz), Rio de Janeiro, Brazil; 3 National Institute of Psychiatry *Ramon de la Fuente Muñiz*, Mexico DF, Mexico; PLOS: Public Library of Science, UNITED STATES

## Abstract

HIV self-testing (HIVST) is an essential tool within the combined HIV prevention package and has been available in Latin America since 2015. However, HIVST use among key populations remains low. This study describes awareness, willingness, and barriers to HIVST among MSM in Brazil, Mexico, and Peru. A cross-sectional web-based survey was advertised in two geosocial networking apps (Grindr and Hornet) and Facebook in 2018. We included cisgender men ≥18 years old who self-reported HIV-negative status. We used multivariable Poisson regression models to calculate adjusted prevalence ratios (aPR) to assess the factors associated with willingness to use HIVST for each country. A total of 18,916 completed the survey, 59% from Brazil, 30% from Mexico, and 11% from Peru. Overall, 20% of MSM had never tested for HIV. Awareness and willingness to use HIVST were higher in Brazil than in Peru and Mexico (p < .001). Across the countries, the patterns of association of willingness with HIVST barriers were similar. Most participants think post-test counseling is essential and that dealing with a positive result would be difficult (aPR 1.13 to 1.37, range of aPRs across the three countries). Having the knowledge to deal with a positive HIVST resulted in increased willingness to use HIVST (aPR range: 1.11 to 1.22), while a lack of trust in HIVST compared to HIV testing in clinics was inversely associated (aPR range: 0.80 to 0.90). In general, willingness to use HIVST was associated with higher income (aPR range: 1.49 to 1.97), higher education (aPR range: 1.13 to 1.42), and willingness to use PrEP (aPR range: 1.19 to 1.72). Efforts to increase HIVST knowledge and resolve perceived barriers are warranted, especially among MSM with lower income and education from Brazil, Mexico, and Peru. Personalized virtual counseling could be crucial among this population. In addition, those willing to use HIVST are also willing to use PrEP. It indicates that HIVST delivery could be incorporated into PrEP programs within the Brazilian Public Health System and eventually in Mexico and Peru.

## Introduction

In 2020, around 38 million people were estimated to be living with HIV globally, 16% of whom remain unaware of their HIV-positive status [1]. Since 2010, Latin America has shown an increasing trend in new HIV infections among adults. In 2019, there were an estimated 100,000 (95% CI 66,000–150,000) new HIV infections and 2.1 million (95% CI 1.4–2.8) people living with HIV in Latin America [1]. HIV incidence is very high among men who have sex with men (MSM) in the region, accounting for 46% of the total new HIV infections in 2020 [1–3]. MSM have an HIV prevalence at least 10-fold higher than the general populations in Brazil, Mexico, and Peru [4–6]. Therefore, this group requires specialized and focused policies, especially MSM with social conditions conferring higher vulnerability [7].

There is consensus about the importance of applying new strategies to make HIV testing services more convenient and attractive for those in need. The aim is to reach the United Nations target of 95% of people living with HIV being aware of their HIV status by 2030 (the first of the UNAIDS 95-95-95 targets). However, Latin America is far from reaching this target; wide-ranging estimates show that around 79% of men living with HIV know their status [1]. In this situation, the HIV self-test (HIVST) could increase HIV diagnosis, paving the way to the first 95-95-95 UNAIDS target [8]. A systematic review of seven RCTs involving MSM found that HIVST increased HIV testing uptake by 1.5 times (RR 1.48; 95% CI: 1.21–1.81) [9].

In 2016 the World Health Organization (WHO) recommended that HIVST be offered as an additional approach to traditional HIV testing services, while in 2019, the WHO recommended social network-based HIV testing approach for key populations as part of the services package [8,10]. In Latin-America, the evidence of HIV self-testing across countries is limited; studies in Brazil [11–15], Mexico [16], Argentina [17] and Peru [11,18] have shown that almost all people surveyed in each study reported they would like to use HIVST. Factors associated with decreased willingness to use HIVST include the high cost of the test [18], being in a relationship, and condomless anal sex in the last three months [16], concern about receiving results alone [12], and social stigma [7,11,19]. However, these studies were applied in particular country settings such as urban or rural areas, or as part of a project that included training to use HIVST, and most of them were conducted prior to the WHO recommendation of HIVST in 2016 [8].

As part of the formative research for The ImPrEP Project (www.imprep.org), in Brazil, Mexico, and Peru, this study aimed to assess awareness, willingness, and barriers to use HIVST among MSM from these countries using a uniform web-based survey.

## Methods

### Study design

We conducted a cross-sectional web-based survey targeting MSM living in Brazil, Mexico, and Peru between March-June 2018. Details of the study design are described elsewhere along with the original analysis results on willingness to use PrEP [14]. Eligibility criteria were: consenting, ≥18-year-old, cisgender men, residents of their respective countries, and self-report HIV-negative status. The questionnaire was programmed on Alchemer^®^ (Brazil) and SurveyMonkey^®^ (Mexico and Peru). We advertised the survey on two geosocial networking (GSN) apps (Hornet^®^ and Grindr^®^) and Facebook^®^. Hornet users received two inbox messages linked to the survey, and Grindr displayed one pop-up advertisement per week to all users for two months. On Facebook^®^, advertisements were targeted at gay and bisexual men. To lessen potential discomfort with sensitive questions, e.g., those related to sexual behavior, answers options included *"I do not want to answer"* or *"I do not know"*. These responses were considered missing for data analysis. Participation was voluntary, and no incentives for participation were provided.

### Variables

#### Outcome

Willingness to use HIVST was defined as the “highest interest” using a five-point Likert scale (strongly disagree, disagree, neutral, agree, and strongly agree) in response to the statement *“I would use the HIV self-test even if I had to pay for it”*, following prior studies accessing willingness to use HIV prevention technologies in Latin America [13,20–22].

#### Socio-demographics

We gathered information about age and self-reported race/skin color. For Brazil and Peru, this was classified as white and non-white (Asian, Black, Indigenous, and Mixed-race), while, in Mexico, response options were indigenous and non-indigenous. Socioeconomic data were measured through monthly income and education. We used the minimum wage (MW) for each country as a measurement scale. In Brazil, we inquired about family monthly income (low ≤3 MW; middle 4–10 MW; and high >10 MW; MW in 2018 was 954 BRL = 250 USD). For Peru and Mexico, the question inquired about personal monthly income. In Peru, it was categorized as low (≤3 MW), middle (4–10 MW), and high (≥10 MW); (MW 2018 was 850 PEN = 265 USD). For Mexico, this was classified as low <3; middle 3–4; high ≥5 (MW 2018 was 2686 MXN = 141 USD). Details of income stratification and rationale are described elsewhere [23]. Education level was dichotomized into less than high school versus high school graduate or higher education. Recruitment was dichotomized in GSN apps (Grindr and Hornet) vs. Facebook.

#### Sexual behavior

Participants were asked about sexual attraction (men only, women only, both men and women) and having a steady partner. In addition, we measured the frequency of using apps for sex and categorized as never, sometimes (once a month, once a week, only on weekends), and daily. HIV perceived risk was measured with the question *“In your opinion*, *what is your risk of getting HIV in the next year*?*”* and dichotomized into “low” and “high” following previous studies [20,24]. The risk of HIV infection was assessed using the HIV Incidence Risk for MSM (HIRI-MSM) scale [25,26], used in previous analyses among MSM in Latin America [13,22,27]. The HIRI-MSM was calculated based on sexual behavior over the last six months (number of partners, condomless receptive anal intercourse, sex with an HIV-positive partner, and use of stimulants). If the participant scored ≥10 points on this scale, they were considered high risk; otherwise, they were deemed low risk. Participants were also asked about sex under the influence of alcohol or drugs (‘Chemsex’) and if they had sex in exchange for money or gifts each during the previous six months.

#### HIV testing, awareness of biomedical HIV prevention, STI testing

Participants were asked about previously testing for HIV with possible answers: “never,” “last three months,” “last 6 months”, “last 12 months”, “more than 12 months,” and “never.” For those who answered *never*, we inquired about reasons for not testing, including options such as fear of a positive result, self-perception of not being at risk for HIV infection, shame of doing the test and reasons related to reluctance to go to the clinic.

We measured the use of HIV pre-and post-exposure prophylaxis (PrEP and PEP). First, PrEP awareness was assessed by asking if they previously heard of PrEP. Then, a brief description of PrEP was provided before questions about PrEP willingness, assessed using a five-point Likert scale of willingness to take daily PrEP (1 = highly unlikely to 5 = highly likely). Thus, PrEP willingness was defined as anyone answering “highly likely.” Furthermore, we asked about previous sexually transmitted infections (STI) diagnoses -gonorrhea, chlamydia, and syphilis- in the last six months [13,14].

#### Awareness of and barriers to HIVST

To assess HIVST awareness, participants were asked if they ever heard about HIVST for HIV diagnosis or not. Independent of their awareness, the questionnaire showed a paragraph explaining HIVST, the need for a confirmatory laboratory test in case of an HIVST positive result, and information about the immunological window period for HIVST. After this explanation, the survey inquired about their willingness to use HIV self-testing.

Barriers to HIVST were assessed with a five-point Likert scale (i.e., strongly disagree, disagree, neutral, agree, and strongly agree). The statements were related to fear (e.g., afraid of doing the self-test alone), shame (e.g., if someone knew that participant uses an HIVST), concerns (about potential HIVST positive result), and trust in using an HIVST (compared to clinic HIV test). The questionnaire also inquired about the best way to provide counseling after testing and personal support to the user who receives an HIVST. The response options were a toll-free telephone call, online chat, mobile app, or clinic visit. We also asked what personal information they would be willing to provide if there was a website where they could register to order an HIVST.

### Statistical analysis

**Fig 1:**
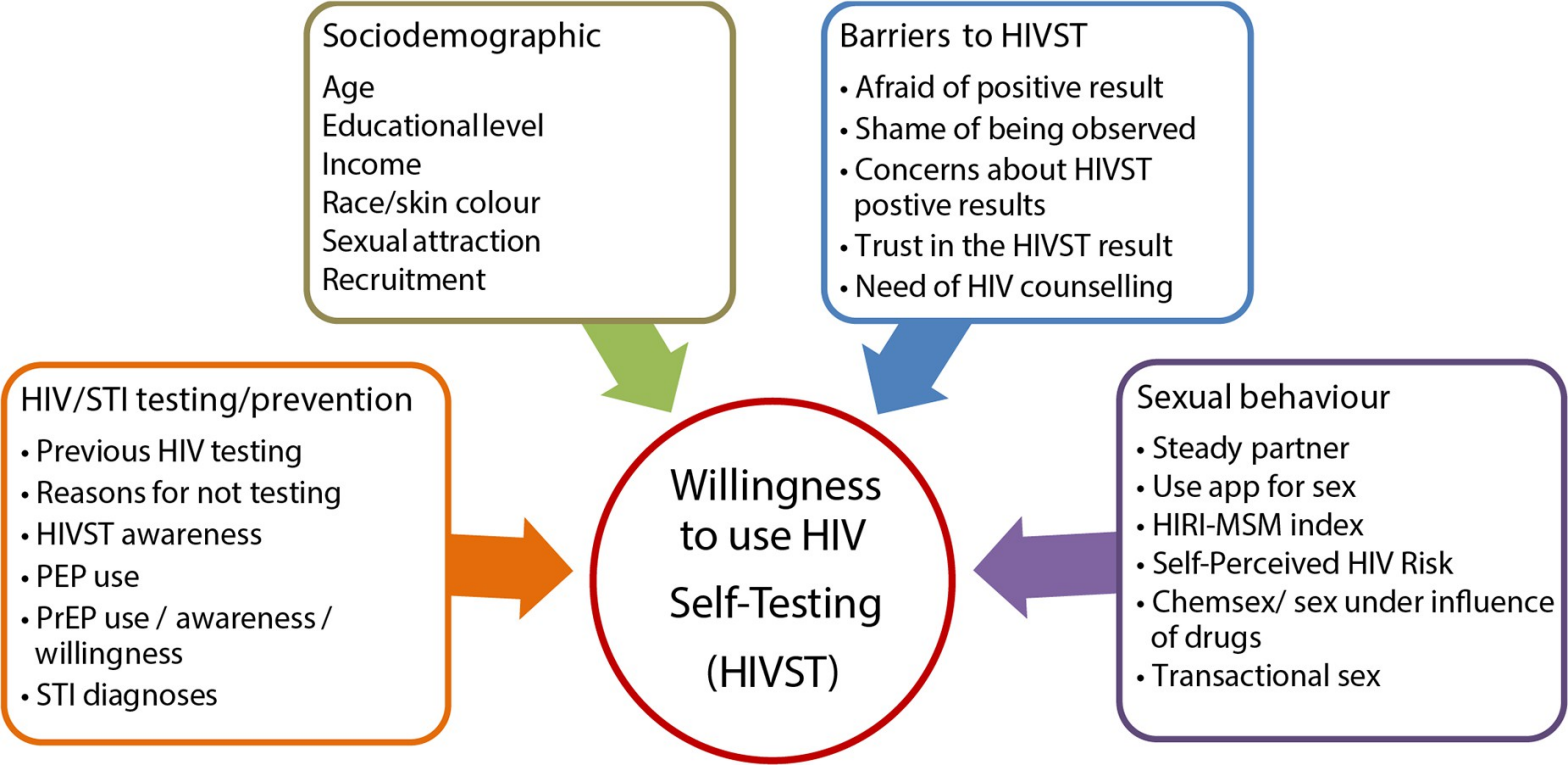
Potential factors associated with willingness to use HIVST.

Bivariate and multivariable modeling was performed using generalized linear modeling (GLM) with Poisson regression and robust adjustment. Poisson regression with robust adjustment was used to obtain efficient results closest to the Mantel-Hansel prevalence ratio [28]. Results are presented as prevalence ratios (PR), adjusted prevalence ratios (aPR), and 95% confidence intervals (CI). Bivariate analysis was performed to evaluate the association between willingness to use HIVST and sociodemographic, sexual behavior, HIV/STI testing/prevention, and barriers to HIVST for each country (Fig 1).

The final multivariable model for each country included variables defined *a priori* as confounders, irrespective of their statistical significance, such as age, education level, monthly income, and race (only in Brazil). Then, additional variables were assessed for inclusion in models for each country using backward stepwise selection; variables with a bivariate p-value<0.01 were included in the initial model and then eliminated if their multivariate p-value was >0.05. Backward elimination started with the least significant variable and then rerunning the model to repeat the process. The final models only kept variables that remained significant (p<0.05) after the deletion process, but included all *a priori* defined cofounders. Statistical analysis was performed using R version 4.2.0 (www.r-project.com).

### Ethical Issues

The ethics committee approved this study in each country. In Brazil, it was obtained from the IRB of INI Evandro Chagas-FIOCRUZ (#CAAE 82021918.0.0000.5262); in Mexico, the research ethics committee of the National Institute of Psychiatry Ramón de la Fuente Muñiz (#CEI/C/038/2018); in Peru, the Research Ethics Committee of Universidad Peruana Cayetano Heredia (#101460). All participants provided their online informed consent before answering the survey, which was completed anonymously.

## Results

### Study population

**Fig 2:**
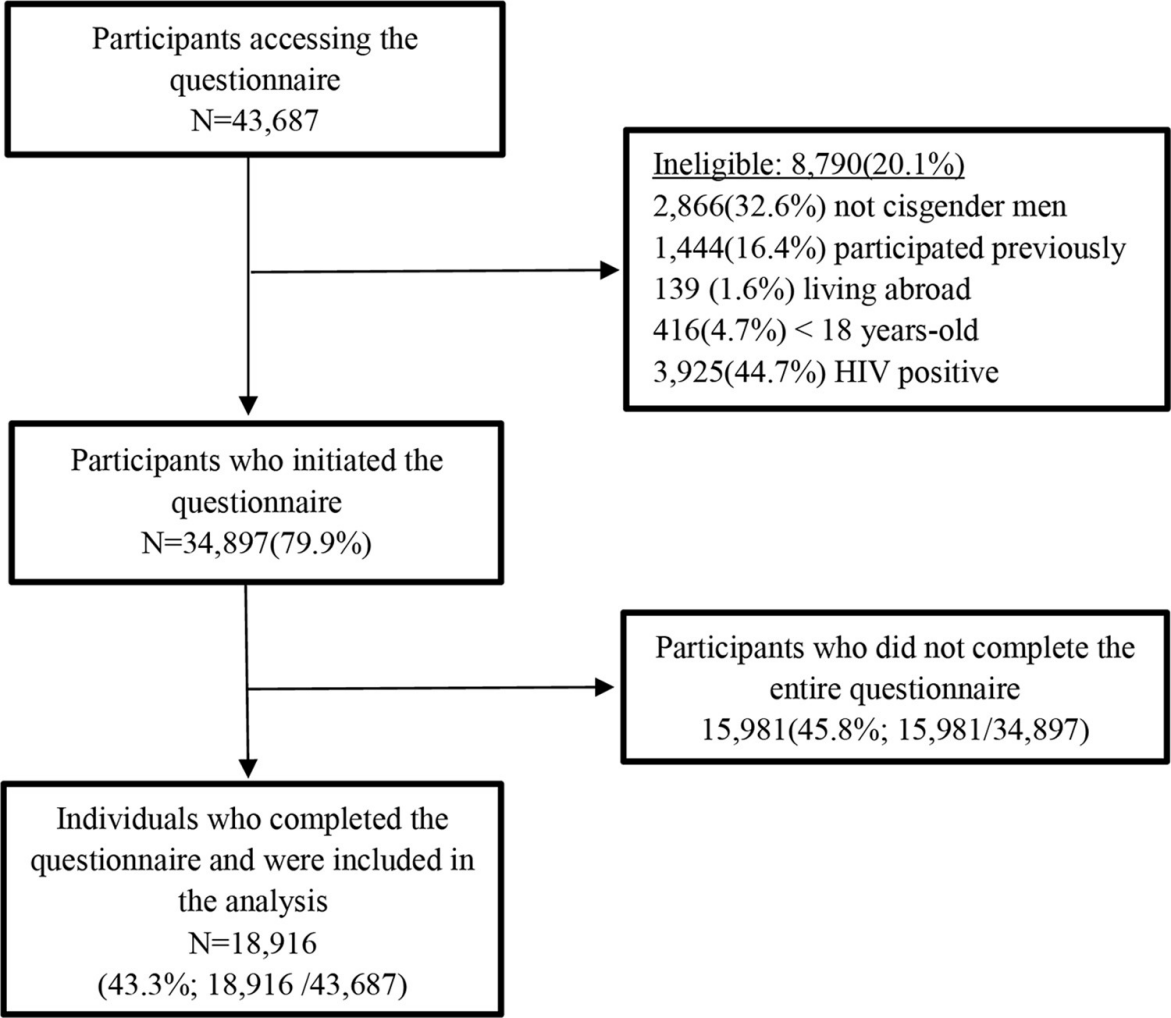
Study flow-chart. Brazil, Mexico, and Peru, 2018.

A total of 43,687 respondents provided informed consent, around 80% (n = 34,897) were eligible to participate, and 43.3% (18,916) completed the survey and were included in the study (Fig 2). As shown in Table 1, of the total eligible, 58.8% (11,118) were from Brazil; 30.3% (5,724) from Mexico; and 11.0% (2,074) from Peru. Nearly half of participants (46.2%), were from the principal cities of each country: São Paulo 16.4% (3,198), Mexico City 13.5% (2,618), Rio de Janeiro 8.2% (1,589), and Lima 8.1% (1,576). The median age was 28 years (IQR 24–34). Race/skin color was differently distributed in each country, reflecting its population’s characteristics. In Brazil, 47.2% were non-white, while in Peru 80.5% were non-white. Only 104 (1.8%) of Mexicans self-reported as Indigenous. Of the total participants, 67.9% had a high school or higher education, and nearly half of individuals (43.3%) were classified as middle income. In Mexico and Brazil, the majority were recruited from advertisements in GSN apps, and in Peru from Facebook.

**Table 1:** Sociodemographic and Sexual Behavior of MSM from Brazil, Mexico, and Peru, 2018.

	Total N = 18916 (100%)	Brazil N = 11118 (58.8%)	Mexico N = 5724 (30.3%)	Peru N = 2074 (11.0%)
**Sociodemographic**				
Age (years) (n = 18915)				
Median (IQR)	28 (24–34)	29 (24–35)	28 (24–34)	26 (22–31)
18–24	5698 (30.1)	3146 (28.3)	1702 (29.7)	850 (41.0)
25–35	9085 (48)	5272 (47.4)	2880 (50.3)	933 (45.0)
≥ 25	4132 (21.8)	2699 (24.3)	1142 (20.0)	291 (14.0)
Race/Skin color (n = 13118)				
White	6263 (47.7)	5873 (52.8)	NA	390 (19.5)
Non-white ^a^	6855 (52.3)	5245 (47.2)	NA	1610 (80.5)
Monthly income ^b^ (n = 18148)				
Low	7155 (39.4)	4990 (44.9)	1484 (28.7)	681 (36.7)
Middle	7855 (43.3)	4627 (41.6)	2316 (44.7)	912 (49.2)
High	3138 (17.3)	1501 (13.5)	1376 (26.6)	261 (14.1)
Education level (years) (n = 18788)				
< High school	6033 (32.1)	4259 (38.6)	1332 (23.3)	442 (21.6)
≥ High school	12755 (67.9)	6761 (61.4)	4392 (76.7)	1602 (78.4)
Recruitment (n = 18916)				
Apps	15,998 (84.6)	9,895 (89.0)	5,184 (90.5)	919 (44.3)
Facebook	2,918 (15.4)	1,223(11.0)	540 (9.5)	1155 (55.7)
**Sexual Behavior**				
Sexual attraction (n = 18862)				
Only men	16837 (89.3)	10163 (91.6)	5032 (88.2)	1642 (79.7)
Men and women	2025 (10.7)	934 (8.4)	673 (11.8)	418 (20.3)
Steady partner(n = 18759)				
No	13804 (73.6)	8191 (74.2)	4165 (73.3)	1448 (71.1)
Yes	4955 (26.4)	2853 (25.8)	1514 (26.7)	588 (28.9)
Use of apps to find sexual partners (n = 18913)				
Never	1611 (8.5)	812 (7.3)	412 (7.2)	387 (18.7)
Sometimes	8499 (44.9)	4286 (38.6)	3214 (56.1)	999 (48.2)
Daily	8803 (46.5)	6017 (54.1)	2098 (36.7)	688 (33.2)
HIV Perceived risk^c^ (n = 18436)				
Low	11953 (64.8)	7,570 (70.1)	3218 (57.3)	1165 (57.4)
High	6483 (35.2)	3223 (29.9)	2396 (42.7)	864 (42.6)
HIRI-MSM ^d^ (n = 18916)				
<10 points; low risk	8989 (47.5)	5269 (47.4)	2774 (48.5)	946 (45.6)
≥10 points; high risk	9927 (52.5)	5849 (52.6)	2950 (51.5)	1128 (54.4)
Sex under the influence of alcohol (n = 18875)				
No	11994 (63.5)	6978 (63.0)	3695 (64.6)	1321 (63.8)
Yes	6881 (36.5)	4105 (37.0)	2027 (35.4)	749 (36.2)
Chemsex^e^ (n = 18861)				
No	15748 (83.5)	9122 (82.4)	4792 (83.8)	1834 (88.6)
Yes	3113 (16.5)	1954 (17.6)	923 (16.2)	236 (11.4)
Transactional sex^e^ (n = 18915)				
No	17901 (94.6)	10515 (94.6)	5461 (95.4)	1925 (92.8)
Yes	1014 (5.4)	602 (5.4)	263 (4.6)	149 (7.2)

Note:

^a^ Asian, Black, Indigenous, and Mix-race

^b^ For Brazil, we considered the number of minimum wages (MW) in the family monthly income: Low < 3 MW; middle 4–10 MW; high > 10 MW (MW in 2018 was 954 BRL = 250 USD, currency from June 2018). For Peru and Mexico, we considered individual monthly income: Low < 3 MW; middle 4–10 MW; high > 10 MW (MW in June 2018 was 850PEN = 265USD [Peru] and 5302MXL = 261USD [Mexico])

^c^ In the coming year

^d^ The HIV Incidence Risk Index for MSM (HIRI-MSM) scale was calculated based on sexual behavior in the previous six months (number of partners, condomless receptive anal intercourse, sex with HIV+ partner and use of stimulants; if ≥10 points, PrEP is recommended); “Unknown” answers scored 0 points in the HIV Incidence Risk for MSM scale.

^e^ During the previous 6 months.

Most participants, 89.3%, reported sexual attraction for men only and 26.4% had a steady partner. The frequency of daily use of apps for sex was higher in Brazil (54.1%), while in Mexico and Peru lower (36.7% and 33.2%, respectively). Sex under the influence of alcohol was proportionally similar among the three countries (overall, 36.5%), but the chemsex was lower in Peru (11.4%), compared to Brazil (17.6%) and Mexico (16.2%). Transactional sex was somewhat higher in Peru (7.2%) compared to Brazil (5.4%) and Mexico (4.6%). During the previous six months, the self-reported prevalence of STI diagnosis was 13.3% in Brazil, 9.6% in Peru, and 6.6% in Mexico. In Brazil, high HIV risk perception reached 29.9%, whereas in Mexico and Peru was similar (~42.6%). However, according to the HIRI-MSM, all participants had similar scores; overall, 52.5% reached the cut-off score (≥10 points) for high HIV risk (Table 1).

Overall, 19.8% never tested for HIV with the highest proportion in Peru (24.2%), while Brazil had the highest proportion of participants who reported an HIV test within the past three months (31.1%). The main reasons for not getting an HIV test were *“I feel afraid of a positive result”* (28.4%), *“I am not at risk of getting infected”* (22.2%), *“I feel ashamed to get tested”* (21.3%). The proportion of MSM aware of HIVST was 35.0%, and the lowest proportion was in Peru (24.8%), followed by Mexico (27.9%) and Brazil (41.0%). Willingness to use HIVST showed similar proportions and order: Brazil, 43.7%; Mexico, 36.3%; Peru, 32.1% (Table 2).

**Table 2:** HIV/STI testing and prevention, including HIVST among MSM from Brazil, Mexico, and Peru, 2018.

	Total N = 18916 (100%)	Brazil N = 11118 (58.8%)	Mexico N = 5724 (30.2%)	Peru N = 2074 (11.0%)
**HIV/STI testing and prevention**				
HIV testing (n = 18780)				
Last 3 months	5333 (28.4)	3436 (31.1)	1368 (24.1)	529 (25.7)
Last 6 months	3248 (17.3)	1942 (17.6)	994 (17.6)	312 (15.2)
Last 12 months	2878 (15.3)	1790 (16.2)	818 (14.4)	270 (13.1)
More than 12 months	3606 (19.2)	1945 (17.6)	1211 (21.4)	450 (21.9)
Never	3715 (19.8)	1940 (17.6)	1277 (22.5)	498 (24.2)
Reasons for not testing^a^ (n = 3469)				
“I feel afraid of a positive result”	1037 (28.4)	448 (23.1)	431 (35.5)	158 (32.3)
“I’m not at risk of getting infected”	809 (22.2)	411 (21.2)	295 (24.3)	103 (21.1)
“I feel ashamed to get tested”	775 (21.3)	423 (21.7)	233 (19.2)	119 (24.3)
“I think it is not practical going to the clinic” or “I feel lazy”	633 (17.4)	400 (20.6)	152 (12.5)	81 (16.6)
“Other”	393 (10.8)	261 (13.4)	104 (8.6)	28 (5.7)
PEP use, last 12 months (yes; n = 18855)	1605(8.5)	1266 (11.4)	267 (4.7)	72 (3.5)
PrEP				
Awareness (yes; n = 18857)	12304 (65.2)	7641 (69.0)	3688 (64.6)	975 (47.2)
Willingness to use (yes; 18916)	12187 (64.4)	6961 (62.6)	4027 (70.4)	1199 (57.8)
Ever used PrEP (yes; n = 18894)	383 (2.0)	258 (2.3)	84 (1.5)	41 (2.0)
STI diagnosis, last 6 months (yes; n = 18349)	1996 (10.9)	1447 (13.3)	362 (6.6)	187 (9.6)
**HIVST**				
Awareness (yes; n = 18678)	6578 (35.2)	4488 (41.0)	1582 (27.9)	508 (24.8)
Willingness to use (yes; n = 18916)	7609 (40.2)	4864 (43.7)	2080 (36.3)	665 (32.1)
Best way to provide counseling/support after HIVST (n = 17891)				
Toll-free telephone number	5693 (31.8)	3417 (32.5)	1627 (30.3)	649 (32.7)
Online chat	1492 (8.3)	769 (7.3)	521 (9.7)	202 (10.2)
App	4322 (24.2)	2479 (23.6)	1289 (24.0)	554 (27.9)
Clinic visit	6384 (35.7)	3863 (36.7)	1940 (36.1)	581 (29.3)
Which personal data would you provide to order an HIVST? (yes, n = 18573)				
Name	12173 (65.5)	7810 (70.8)	3414 (61.8)	949 (47.1)
National Identification Number	7839 (42.2)	5555 (50.3)	1518 (27.5)	766 (38.0)
e-mail	13492 (72.6)	8780 (79.6)	3662 (66.3)	1050 (52.1)
Mobile number	13515 (72.8)	8222 (74.5)	3891 (70.5)	1402 (69.5)
Facebook profile	2438 (13.1)	1443 (13.1)	721 (13.1)	274 (13.6)
Landline number	2516 (13.5)	1758 (15.9)	645 (11.7)	113 (5.6)
I would provide no data	1353 (7.3)	811 (7.4)	363 (6.6)	179 (8.9)
Other	342 (1.8)	257 (2.3)	56 (1.0)	29 (1.4)

^a^Only those who reported never tested for HIV.

Participants preferred post-HIVST counseling via toll-free calls by support (31.8%) or by clinic visits (35.7%). The personal data that participants would provide to get an HIVST via website showed different concerns about privacy information. Brazil had the highest percentage of participants willing to give their names (70.8%), while in Mexico was 61.8%, and in Peru 47.1%. Furthermore, Mexico had the lowest proportion of participants who would give their national identification number (27.5%), whereas in Brazil this result reached 50.3%, and Peru 38.0%. On the other hand, similar proportions of participants from the three countries agreed on providing a cell phone number (72.8%). Similarly, a small percentage said that they would not provide any information (7.3%).

**Fig 3:**
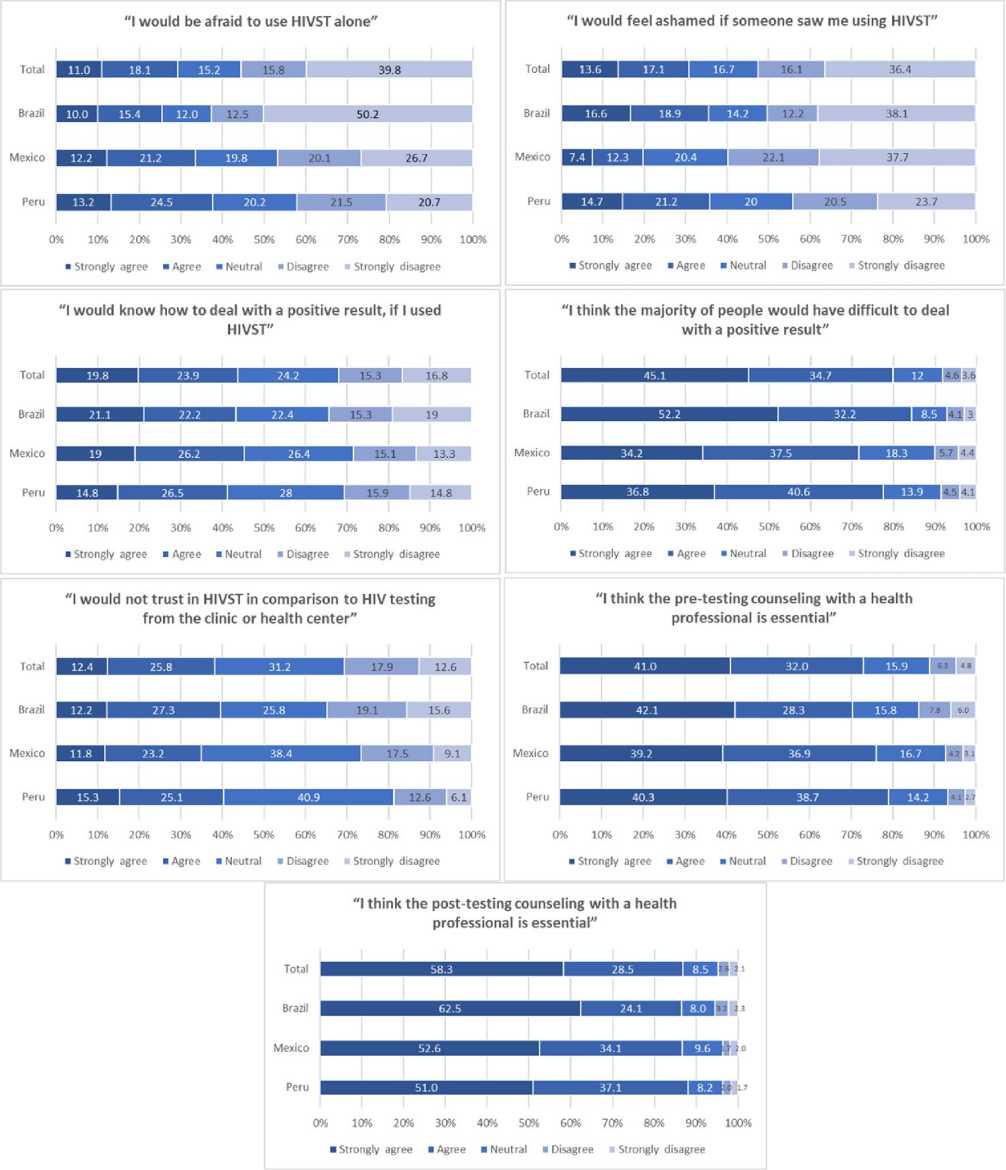
Barriers to use HIVST among MSM in Brazil, Mexico, and Peru, 2018.

Barriers related to use HIVST according to each country are depicted in Fig 3. The most reported barrier was the post-HIVST counselling from a health professional. Additionally, most of the participants strongly agreed or agreed with the need for pre and post-HIVST counselling (73.0% and 86.8%, respectively). Another critical barrier was the difficulty of dealing with an HIVST positive result according to 80.1% of participants who strongly agreed or agreed. In contrast, less than half (43.8%) strongly agreed or agreed on the statement, *“I would know how to deal with a positive result if I used HIVST*.*”* Other reported barriers that participants strongly agreed or agreed were the shame if someone saw they use HIVST (30.6%), and the fear of using HIVST alone (29.0%). Lastly, 38.2% strongly agreed or agree regarding the statement related to trust in HIVST: “I would not trust an HIVST in comparison to HIV testing from the clinic or health center”.

### Factors associated with willingness to use HIVST

In the adjusted multivariate models (Table 3), reporting high monthly income (aPR: 1.49 to 1.97, range of aPRs across the three countries) and high educational level (aPR range: 1.13 to 1.42) were associated with willingness to use HIVST for the three countries, and recruitment through GSN apps only for Brazil (aPR: 1.11, 95%CI 1.00–1.23). Regarding sexual behavior, in Brazil high score in HIRI-MSM scale (aPR: 1.06, 95%CI 1.00–1.14), the use of apps for sex in Peru (aPR 1.43, 95%CI 1.17–1.75), and sex under the influence of alcohol in Mexico (aPR 1.13, 95%CI 1.01–1.27) were associated with willingness to use HIVST. Likewise, willingness to use PrEP showed a positive association with willingness to use HIVST in all three countries (aPR range: 1.19 to 1.72). In contrast, transactional sex in Brazil (aPR 0.85, 95%CI 0.73–0.99) and HIVST awareness in Mexico (aPR 0.80, 95%CI 0.70–0.91) were inversely associated with willingness to use HIVST.

**Table 3:** Factors associated with willingness to use HIVST among MSM from Brazil, Mexico, and Peru, 2018.

	Brazil		Mexico		Peru		
Variable	Crude PR (95% CI)	Adjusted aPR (95% CI)^a^	Crude PR (95% CI)	Adjusted aPR (95% CI)^a^		Crude PR (95% CI)	Adjusted aPR (95% CI)^a^
**Sociodemographic**							
Age (years)							
18–24	**0.80**	0.94 (0.86–1.02)	**0.89**	1.11 (0.97–1.27)		**0.80**	1.22 (0.97–1.54)
≥ 25 years	**Ref.**	Ref.	**Ref.**	Ref.		**Ref.**	Ref.
Race/skin color							
White	**Ref.**	Ref.	NA	NA		Ref.	b
Non-white ^c^	**0.91**	1.00 (0.93–1.06)	NA	NA		1.03	B
Monthly income^d^							
Low	**Ref.**	Ref.	**Ref.**	**Ref.**		**Ref.**	**Ref.**
Middle	**1.34**	**1.33 (1.23–1.43)**	**1.17**	**1.15 (1.00–1.33)**		**1.20**	**1.10 (0.86–1.39)**
High	**1.66**	**1.79 (1.62–1.99)**	**1.41**	**1.49 (1.26–1.76)**		**1.72**	**1.97 (1.41–2.75)**
Educational level							
< High school	**Ref.**	Ref.	Ref.	**Ref.**		**Ref.**	**Ref.**
≥ High school	**1.34**	**1.24 (1.15–1.33)**	1.19	**1.13 (1.00–1.31)**		**1.43**	**1.42 (1.07–1.88)**
Recruitment							
GSN App	**1.08**	**1.11 (1.00–1.23)**	0.96	b		1.08	b
Facebook	**Ref.**	Ref.	Ref.	b		Ref.	b
**Sexual behavior**							
Sexual attraction							
Men	1.03	b	1.10	b		1.07	b
Men and women	Ref.	b	Ref.	b		Ref.	b
Steady partner (yes vs. no)							
Yes	**1.06**	b	1.01	b		1.00	b
No	**Ref.**	b	Ref.	b		Ref.	b
Use apps for sex							
Never/sometimes	Ref.	b	Ref.	b		**Ref.**	**Ref.**
Daily	1.03	b	1.11	b		**1.29**	**1.43 (1.17–1.75)**
HIV Perceived risk^e^							
Low	Ref.	b	Ref.	b		Ref.	b
High	1.02	b	1.06	b		1.06	b
HIRI-MSM scale ^f^							
Low	**Ref.**	Ref.	Ref.	b		Ref.	b
High	**1.05**	**1.06 (1.00–1.14)**	1.07	b		1.00	b
Sex under the influence of alcohol ^g^							
Yes	1.04	b	**1.12**	**1.13 (1.01–1.27)**		1.10	b
No	Ref.	b	**Ref.**	**Ref.**		Ref.	b
Chemsex ^g^							
Yes	1.00	b	1.09	b		1.01	b
No	Ref.	b	Ref.	b		Ref.	b
Transactional sex ^g^							
Yes	**0.80**	**0.85 (0.73–0.99)**	1.13	b		1.14	b
No	**Ref.**	Ref.	Ref.	b		Ref.	b
STI diagnoses							
Yes	0.99	b	1.06	b		1.00	b
No	Ref.	b	Ref.	b		Ref.	b
**HIV/STI testing and prevention**							
HIV testing							
At least once lifetime	1.11	b	0.98	b		1.17	b
Never	Ref.	b	Ref.	Ref.		Ref.	b
HIVST awareness							
Yes	1.05	b	**0.93**	**0.80 (0.70–0.91)**		0.90	b
No	Ref.	b	**Ref.**	**Ref.**		Ref.	b
PEP use							
Yes	0.99	b	1.06	b		1.18	b
No	Ref.	b	Ref.	b		Ref.	b
PrEP awareness							
Yes	**1.10**	b	1.08	b		**1.27**	b
No	**Ref.**	b	Ref.	b		**Ref.**	b
Willingness to use PrEP							
Yes	**1.21**	**1.19 (1.12–1.28)**	**1.45**	**1.44 (1.27–1.63)**		**1.68**	**1.72 (1.40–2.11)**
No	**Ref.**	Ref.	**Ref.**	**Ref.**		**Ref.**	**Ref.**
Ever used PrEP							
Yes	0.86		1.19	b		1.15	b
No	Ref.	Ref.	Ref.	b		Ref.	b
**Barriers to HIVST**							
“I would not trust in HIVST in comparison to HIV testing from the clinic or health center”	**0.91**	**0.90 (0.88–0.93)**	**0.87**	**0.80 (0.76–0.84)**		**0.87**	**0.82 (0.74–0.90)**
“I would be afraid to use HIVST alone”	**0.90**	**0.91 (0.88–0.93)**	**0.92**	b		**0.89**	**0.92 (0.85–0.99)**
“I would feel ashamed if someone knew I am using an HIVST”	0.99	b	**0.90**	**0.96 (0.92–0.99)**		**0.93**	b
“I would know how to deal with a positive result, if I used HIVST”	**1.10**	**1.11 (1.08–1.14)**	**1.18**	**1.21 (1.15–1.27)**		**1.20**	**1.22 (1.12–1.33)**
“I think the majority of people would have difficult to deal with a positive result”	**1.03**	**1.09 (1.05–1.14)**	**1.06**	**1.08 (1.02–1.14)**		**1.07**	**1.14 (1.03–1.27)**
“I think the pre-test counseling with a health professional is essential”	**0.94**	**0.95 (0.91–0.98)**	**1.06**	b		1.02	b
“I think the post-test counseling with a health professional is essential”	**1.02**	**1.13 (1.08–1.18)**	**1.29**	**1.37 (1.29–1.48)**		**1.18**	**1.33 (1.16–1.52)**

Barriers to HIVST were also associated with willingness to use HIVST in the final multivariate models. Among the three countries, knowing how to deal with an HIV positive result was associated with willingness to use HIVST (aPR range: 1.11 to 1.20), while not trusting in HIVST in comparison to HIV testing from clinic or health center was associated with lower willingness to use HIVST (aPR range: 0.80 to 0.90). In Brazil and Mexico, being afraid to use HIVST alone was associated with lower willingness to use HIVST (aPR range: 0.89 to 0.91); in Mexico, feeling ashamed if someone knew you were using HIVST showed a similar effect (aPR: 0.96, 95%CI 0.92–099). Lastly, thinking that post-test counseling with a health professional is essential was associated with higher willingness to use HIVST in all countries (aPR range: 1.13 to 1.37). In contrast, indicating an importance of pre-test counseling was associated with lower willingness to use HIVST only in Brazil (aPR 0.95, 95%CI 0.91–0.98).

## Discussion

This study provides information on awareness, willingness to use, and barriers related to HIVST among a large sample of MSM from Brazil, Mexico, and Peru. Our results suggest that approximately 35% of participants in Peru and Mexico and 40% in Brazil were aware and willing to use HIVST. Across the countries, willingness to use HIVST was associated with higher income, higher education, and willingness to use PrEP. Existing pre- and pos-test counseling with a health professional and difficulties of dealing with an HIVST positive result were the main endorsed HIVST barriers in the three countries.

Our regression models suggest some factors associated with willingness to use HIVST that are common in Brazil, Mexico, and Peru and other factors that are only significant for one or two countries. Socioeconomic indicators, such as higher income and education level [11,29–32], were directly related to willingness to use HIVST, statistically significant for all three countries. These relationships could reflect that more years of education could lead to a higher knowledge of new self-diagnostic methods [33,34]. Likewise, purchasing power would be a precondition related to willingness to get an HIV self-test kit [35,36] in countries where HIVST is not available free of charge through the Public Health System, as in Brazil. Although we did not evaluate it here, other studies have shown that the HIVST kit’s price plays a critical role in its use [18]. Also, our findings suggest that sexual health education approach including HIVST might be crucial among key populations [37–39], since poverty, lack of schooling along with existing social inequities are barriers to HIV services that could have been increased during and after the COVID-19 pandemic [1].

Willingness to use PrEP was directly associated with willingness to use HIVST, and it was statistically significant in the final model for all three countries. This positive relationship could be explained since PrEP and HIVST have a common target population and interest in both may represent individuals open to innovation in HIV prevention. This finding suggests that HIVST and PrEP could be offered together and delivered through the same platform. Previous studies have made both services available through a mobile app [40,41], while other pilot studies have jointly offered PrEP and HIVST [42,43], with an increasing in testing. Telemedicine procedures and HIVST distribution were implemented in Brazilian PrEP services since the onset of the Covid-19 pandemic [44], and the acceptability of both strategies was very high [45]. Future research in Latin America could explore the interaction of PrEP and HIVST to improve PrEP adherence and testing frequency, likewise a better understanding of the demand for these services.

The relationship of sexual behavior characteristics with willingness to use HIVST differed by country. MSM at higher risk for HIV (measured by HIRI-MSM scale) and sex under the influence of alcohol was associated with higher willingness to use HIVST in Mexico and Brazil, respectively. Both results are promising and indicate that MSM with a high vulnerability for HIV may be more interested in HIVST. Conversely, individuals reporting transactional sex, who maybe have higher vulnerability to HIV, had reduced willingness to use HIVST in Brazil. In our study, the proportion of Brazilian MSM who strongly agreed or agreed with the statement *“I would be afraid to use HIVST alone”* was higher among those reporting transactional sex vs. not reporting (29.4% vs. 25.1%; p = 0.019), which could partially explain our findings. Daily use of apps for sex was only associated with willingness to use HIVST in Peru. Thus, in Peru, these apps could be segmented to a population group more adapted to the use of new technologies such as HIVST. However, the use of apps for sex is lower in Peru than in the remaining countries: 18.7% of Peruvians had never used an app for sex, whereas in Brazil and Mexico, that answer was only 7%. Likewise, in Peru, participants were principally recruited from Facebook 55.7%, while in Brazil and Mexico, almost 90% were reached through GSN apps (Grindr and Hornet). The latter result could make it more difficult to find differences in the adjusted model for willingness to use HIVST among those who use and do not use these apps in Brazil and Mexico.

Surprisingly, HIVST awareness was associated with lower willingness to use HIVST in the adjusted model for Mexico. This could be a sign that a communication campaign to clarify doubts and fears about HIVST would be crucial, especially highlighting the utility of HIVST and its use as a preliminary test that would then require confirmation, but can help identify individuals in need of further testing. The lack of adequate information is a barrier identified and translated into two questions. First, only 27.9% of Mexican MSM from our sample had heard of HIVST before. Second, participants who disagreed with the statement *"I would not trust in HIVST compared to HIV testing from the clinic or health center"* had significantly higher willingness to use HIVST willingness in all three countries. Both results suggest an excellent opportunity for information campaigns, distribution of HIVST through web channels, pharmacies, and for HIV partner notification services.

Indicating the need for post-test counseling had a positive relationship with willingness to use HIVST in all three countries, but the statement about the need for pre-test counseling had an inverse relationship, only in Brazil. The positive association with post-test counseling reveals that there would be tension between preferences for privacy and support [35]. It would be interesting to know if virtual counseling would be an acceptable replacement [46,47]. A real-time conversation with a professional while knowing that the health professional is not observing the participants could be a relief for some participants. It is a possible mechanism since 72.8% of participants agreed to provide their cell phone number to access an HIVST through a website that requires this type of information. Conversely, pre-test counseling may be perceived by Brazilian MSM as an obstacle to obtaining the HIVST kit, or it may be seen as an intrusion to privacy if pre-counseling implies knowing the identity of the person who will use the test.

Regarding barriers, statements related to knowledge about dealing with an HIVST positive result was associated with higher willingness to use HIVST in all three countries. In contrast, fear of using HIVST alone was associated with lower willingness in Brazil and Peru but not in Mexico. Both results confirm the importance of counseling, usually accompanying home-based testing studies [48]. Different non-personal counseling mechanisms have been piloted in urban and rural settings and high-income and low-income countries [49,50]. Virtual counseling represents an excellent opportunity to become a valuable tool to empower people for self-care, reducing the problems associated with HIV/AIDS-related stigmas [7,11,19]. Approximately 20% had never taken an HIV test in our whole sample, and another 20% had not been tested in the last 12 months, which is worrisome considering UNAIDS 95-95-95 targets to end HIV epidemic [1]. The possibility of implementing virtual or telephone counseling would increase the use of HIVST [10,51] and consequently increase HIV testing and diagnoses. It could be helpful since most previously focused MSM services in Brazil, Mexico, and Peru are centered in urban areas and cannot cover all the territory. However, HIVST could be an approach to expanding health services related to HIV and other STIs, as well as increase HIV testing uptake, frequency, and yield without social harms as previously observed in other settings [9,52]. Previous pilot studies conducted in Brazil [53], China [54], Uganda [55], Nigeria [56] and South Africa [57] have shown that HIVST is highly acceptable and feasible to implement among MSM and other populations.

Among limitations, first, the cross-sectional design did not permit causal inference; we only assessed factors associated with willingness to use HIVST. Second, since it is a web-based survey, the sample was not probabilistic, and it is only representative of an MSM population with access to smartphones or computers. Third, the high attrition (45.8%) may preclude the generalizability of our findings, as the characteristics of MSM who do not complete the survey may differ from those who complete it, as seen in previous studies [13,58]. In this study, the MSM sample who did not complete the survey vs. those who completed was mainly composed by Brazilians (64.9% vs. 58.8%; p < .001), aged 18–24 years (38.1% vs. 30.1%; p < .001), non-white (55.0 vs. 51.6%; p < .001), lower income (50.4% vs. 39.4%; p < .001), and lower education (44.1% vs. 32.1%; p < .001). Forth, self-reporting in a web-based survey could introduce a social desirability bias. Finally, the questionnaire’s length was designed to be answered online, which limited the number of questions.

Conversely, the study’s strength lies in the analysis of three Latin American countries with a questionnaire adapted for each setting. This study would be the first joint research among countries in the region to assess willingness to use HIVST. The MSM population was adequately targeted, via Facebook or apps such as Grindr and Hornet, resulting in a worthwhile sample. Furthermore, the privacy of participants to answer the questionnaire could be an advantage over face-to-face surveys.

Finally, it is essential to mention the promising future of HIVST. As a consequence of COVID-19, especially in low- and middle-income countries, the current crisis of public health services lays bare the need for innovative approaches to continue offering HIV testing services while maintaining a physical distance. HIVST could become a valuable tool to keep HIV testing advances achieved in the last years, focused on the UNAIDS target of 95-95-95 to 2030 [51]. Future research in this area needs to understand what factors and strategies could be crucial to delivering HIV-related health services successfully.

## Conclusions

Overall, we identified factors associated with HIVST that were relevant in all three countries. Sociodemographic variables such as income and education level; other prevention variables such as the willingness to use PrEP; and potential barriers that could be a challenge to HIVST expansion, such as the importance of pre- and post-test counseling for HIVST and doubts about how to deal with a positive HIVST result. These findings should promote future research about HIVST, considering the cultural reception, demand characteristics, and other barriers or incentives that could play a crucial role among potential users.

## Supporting information

S1 Data Dataset. (CSV)
